# Identifying Individuals With Mild Cognitive Impairment Using Working Memory-Induced Intra-Subject Variability of Resting-State EEGs

**DOI:** 10.3389/fncom.2021.700467

**Published:** 2021-08-04

**Authors:** Thanh-Tung Trinh, Chia-Fen Tsai, Yu-Tsung Hsiao, Chun-Ying Lee, Chien-Te Wu, Yi-Hung Liu

**Affiliations:** ^1^Neural Engineering and Smart Systems Laboratory, Graduate Institute of Manufacturing Technology, College of Mechanical and Electrical Engineering, National Taipei University of Technology (Taipei Tech), Taipei, Taiwan; ^2^Department of Psychiatry, Division of Geriatric Psychiatry, Taipei Veterans General Hospital, Taipei, Taiwan; ^3^Faculty of Medicine, National Yang Ming Chiao Tung University, Taipei, Taiwan; ^4^Neural Engineering and Smart Systems Laboratory, Graduate Institute of Mechatronic Engineering, National Taipei University of Technology (Taipei Tech), Taipei, Taiwan; ^5^Department of Mechanical Engineering, National Taipei University of Technology (Taipei Tech), Taipei, Taiwan; ^6^International Research Center for Neurointelligence (WPI-IRCN), The University of Tokyo Institutes for Advanced Study (UTIAS), The University of Tokyo, Tokyo, Japan; ^7^Department of Mechanical Engineering, National Taiwan University of Science and Technology (Taiwan Tech), Taipei, Taiwan

**Keywords:** intra-subject variability, electroencephalography, mild cognitive impairment, Alzheimer's disease, between-run similarity, brain-computer interface, machine learning

## Abstract

Individuals with mild cognitive impairment (MCI) are at high risk of developing into dementia (e. g., Alzheimer's disease, AD). A reliable and effective approach for early detection of MCI has become a critical challenge. Although compared with other costly or risky lab tests, electroencephalogram (EEG) seems to be an ideal alternative measure for early detection of MCI, searching for valid EEG features for classification between healthy controls (HCs) and individuals with MCI remains to be largely unexplored. Here, we design a novel feature extraction framework and propose that the spectral-power-based task-induced intra-subject variability extracted by this framework can be an encouraging candidate EEG feature for the early detection of MCI. In this framework, we extracted the task-induced intra-subject spectral power variability of resting-state EEGs (as measured by a between-run similarity) before and after participants performing cognitively exhausted working memory tasks as the candidate feature. The results from 74 participants (23 individuals with AD, 24 individuals with MCI, 27 HC) showed that the between-run similarity over the frontal and central scalp regions in the HC group is higher than that in the AD or MCI group. Furthermore, using a feature selection scheme and a support vector machine (SVM) classifier, the between-run similarity showed encouraging leave-one-participant-out cross-validation (LOPO-CV) classification performance for the classification between the MCI and HC (80.39%) groups and between the AD vs. HC groups (78%), and its classification performance is superior to other widely-used features such as spectral powers, coherence, and the complexity estimated by Katz's method extracted from single-run resting-state EEGs (a common approach in previous studies). The results based on LOPO-CV, therefore, suggest that the spectral-power-based task-induced intra-subject EEG variability extracted by the proposed feature extraction framework has the potential to serve as a neurophysiological feature for the early detection of MCI in individuals.

## Introduction

Alzheimer's dementia has become the most prevalent type of neurodegenerative dementia. There are nearly 10 million new cases of dementia every year worldwide and 60–70% of these new cases are diagnosed with AD (World Health Organization, 2020). The prevalence of AD generally increases with age: the prevalence is 1% for people between 60 and 64 years, but it increases to 38% for people over 85 years (Ferrara et al., 2008). Although mild cognitive impairment (MCI), typically as a transitional state between normal aging and very early dementia, does not usually impact the daily life of individuals (Petersen, 2010), it may convert to AD or other types of dementia with a high risk. For example, a study reported that 15% of MCI in individuals older than 65 years old may develop into dementia (Alzheimer association, 2020), whereas another study reported that MCI of 32% of individuals developed into AD at the 5th-year follow-up (Chen Y. et al., 2020; Alzheimer association, 2021). Early detection and intervention for individuals with MCI will, therefore, be an important strategy in the fight to reduce the impact of AD on our community. However, early detection of MCI is challenging, as older adults with MCI are often not aware of the subtle decline in their cognitive function, which primarily prevents them from seeking medical advice or even interventions.

Several biomarkers have been proposed to help physicians verify the diagnosis of dementia due to AD; In contrast, the diagnosis of MCI heavily relies rather on neuropsychological assessments. For the diagnosis of AD, a common method is to detect human brain amyloid-beta (Aβ) deposition and abnormal aggregation of tau protein. The concentration ratio of Aβ42 to Aβ40 (Aβ42/40 ratio), the concentration of Aβ42 level and positive amyloid, and tau PET scan are considered as important biomarkers to detect AD (Hansson et al., 2019). However, these biomarkers are not ideal solutions for the community health care system, because they are expansive, time-consuming, invasive, and radiational in nature. In addition, although recently, there have been attempts to establish a biomarker-based guideline for the diagnosis of MCI (Ritchie et al., 2014; Martinez et al., 2017; Ross et al., 2021), there is still room for improvement in terms of accessibility, reliability, and validity of these biomarkers. Electroencephalography (EEG), on the other hand, is a promising alternative due to its non-invasive nature and relatively much lower costs. EEGs may, therefore, have great potential for assisting the clinical characterization of MCI and AD (Poza et al., 2014).

Resting-state EEGs, typically recorded while participants are not doing anything purposefully, has become a popular approach in clinical research with the patient population who has short attention span or difficulties performing a goal-directed task. Previously, a large body of literature based on single-session resting-state EEG has found differences in EEG features between AD and HC, such as spectral powers of different frequency bands, complexity, and connectivity. For example, compared with the HC group, individuals with AD showed lower signal complexity (Abasolo et al., 2005, 2006, 2008; Liu et al., 2015), a higher power of slow oscillations (delta, theta) and lower power of fast oscillation (alpha, beta, gamma) over the temporal, parietal, and occipital scalp regions (Huang et al., 2000; Rossini et al., 2007; Roh et al., 2011; Ishii et al., 2017), and lower electrode-to-electrode connectivity (Wang et al., 2014; Engels et al., 2015; Hata et al., 2016) in resting-state EEGs. Furthermore, the entropy-based complexity of EEG signals seems to gradually decrease with disease development (Sun et al., 2020). However, differences in resting-state EEGs between the MCI group and the HC group are relatively less studied. Few studies reported a non-significant trend for loss of complexity in individuals with MCI compared with HC (Park et al., 2007; Dauwels et al., 2011; Labate et al., 2013; Xu and Tao, 2013; Seker et al., 2021). Searching for a more distinguishing EEG signature based on resting-state recordings for the classification between MCI and HC appears to be a critical challenge.

In addition to altered resting-states, memory dysfunction can be another key clinical trait for inducing relevant EEG patterns to discriminate between AD/MCI and HC. Memory dysfunction is one of the critical diagnosis criteria for AD (American Psychiatric Association, 2013), and among all types of memory dysfunction, working memory impairment is often observed in both MCI and AD. Working memory refers to the ability to access and manipulate information that is stored in a short period of time (Baddeley et al., 2015). Most complex cognitive abilities, such as spatial orientation, problem solving, and reading, require working memory functions (Kirova et al., 2015). Specifically, individuals with MCI typically show performance declination in verbal/visual working memory (Saunders and Summers, 2010), sentence span, operation span (Gagnon and Belleville, 2011), digit span, letter-number sequencing, and arithmetic operation (Kessels et al., 2011). Since impaired working memory is commonly observed in individuals with MCI, working memory tasks can be a good candidate to induce task-relevant differences in resting-state EEGs between the MCI group and the HC group.

This study, therefore, aimed to capitalize on the spectral-power-based task-induced intra-subject variability of EEGs recorded in two separate runs of resting-states, before and after a challenging working memory task. Since working memory tasks are presumably more cognitively exhausted for the MCI or AD group than the HC group, we hypothesize that the difference in the neurophysiological patterns of the before-task and after-task “resting-state” in the brain will be larger for the MCI group than the HC group, and such difference carries more discriminative information for classification in comparison with the approach using single-run resting-state EEGs that has been adopted in previous studies related to the MCI-HC classification. To achieve this goal, we designed a novel feature extraction framework in which we introduced the delayed matching-to-sample (DSTM) task as a cognitively challenging behavior test, applied a similarity-based approach (Chen G. et al., 2020) to quantitatively evaluate the task-induced intra-subject variation of resting-state EEG powers, and used it as a neural marker to classify between the MCI and HC groups. To the best knowledge of the authors, this is the first study that focuses on the analysis of task-induced intra-subject variability between two separate runs of resting-state EEGs for the detection of MCI. First, we investigated the group difference in between-run similarity of resting-state EEGs across different frequency bands and scalp regions between the AD, MCI, and HC groups. Second, we used machine-learning based feature selection methods to determine the best combination of intra-subject variability features for MCI-HC classification. The results showed that the proposed novel intra-subject variability feature can be a promising one to further develop an EEG-based computer-aided diagnosis method for the early detection of MCI.

## Method

### Participants

This study included 23 individuals with Alzheimer's disease (AD) (nine females, mean age of 71.65 ± 5.36 y/o), 24 individuals with mild cognitive impairment (MCI) (14 females, mean age of 70.96 ± 8.2 y/o) in the patient group and 27 participants in the healthy control (HC) group (17 females, mean age of 69.93 ± 4.98 y/o). Data collection was conducted from July 2017 to July 2020 at an outpatient memory clinic of a tertiary 2,700-bed referral center (Table 1). The diagnosis of participants from the patient group was based on the results of clinical interviews, neuropsychological examinations, laboratory findings, and image investigations (CT and/or MRI) and was confirmed at clinical consensus meetings by board-certified psychiatrists. The core clinical criteria recommended by National Institute on Aging and the Alzheimer's Association (NIA-AA) (Albert et al., 2011; McKhann et al., 2011) were used for the diagnosis of AD and MCI. Participants from the control group were enrolled *via* advertisement and confirmed as not having any condition for all-cause dementia listed in the NIA-AA criteria. Furthermore, the participants from the control group were all tested with neuropsychological battery, which resulted in the normal range on standardized neuropsychological batteries after adjustment for education (Tsai et al., 2012).

**Table 1:** Demographics and questionnaire data [Mean (SD)].

**Variable**	**HC**	**MCI**	**AD**	***p***	**Effect size**
	***n* = 27**	***n* = 24**	***n* = 23**		
Gender	17 F, 10 M	14 F, 10 M	9 F, 14 M	0.212	0.145
Age	69.93 (4.98)	70.96 (8.20)	71.65 (5.36)	0.621	0.013
Education (years)	13.44 (3.18)	12.13 (3.76)	11.43 (4.35)	0.132	0.050
MMSE	28.26 (1.79)	26.58 (1.89)	21.35 (5.77)	<0.001	0.412
MoCA	25.89 (3.29)	23.08 (4.11)	15.96 (6.43)	<0.001	0.447

*F, female; M, male; HC, healthy controls; MCI, mild cognitive impairment; AD, Alzheimer's disease; MMSE, mini-mental state examination; MoCA, montreal cognitive assessment*.

*Gender: chi-square test of independence*.

*Age/Education year/MMSE/MoCA: one-way ANOVA*.

The exclusion criteria for all three groups were: (1) recent major psychiatric comorbidity (clinically diagnosed in the 6 months prior to the current neuropsychological evaluation), (2) motor and/or sensory deficits that constituted confounding variables in the assessment of cognitive functions, and (3) neurological illness or condition that may affect cognition.

The study protocol was reviewed and approved by the institutional review board of Taipei Veterans General Hospital (IRB No: 2017-06-009A). Before the experiment, written informed consents were obtained from all the participants or their legally authorized representatives according to the Declaration of Helsinki.

### Experimental Procedure

**Figure 1:**
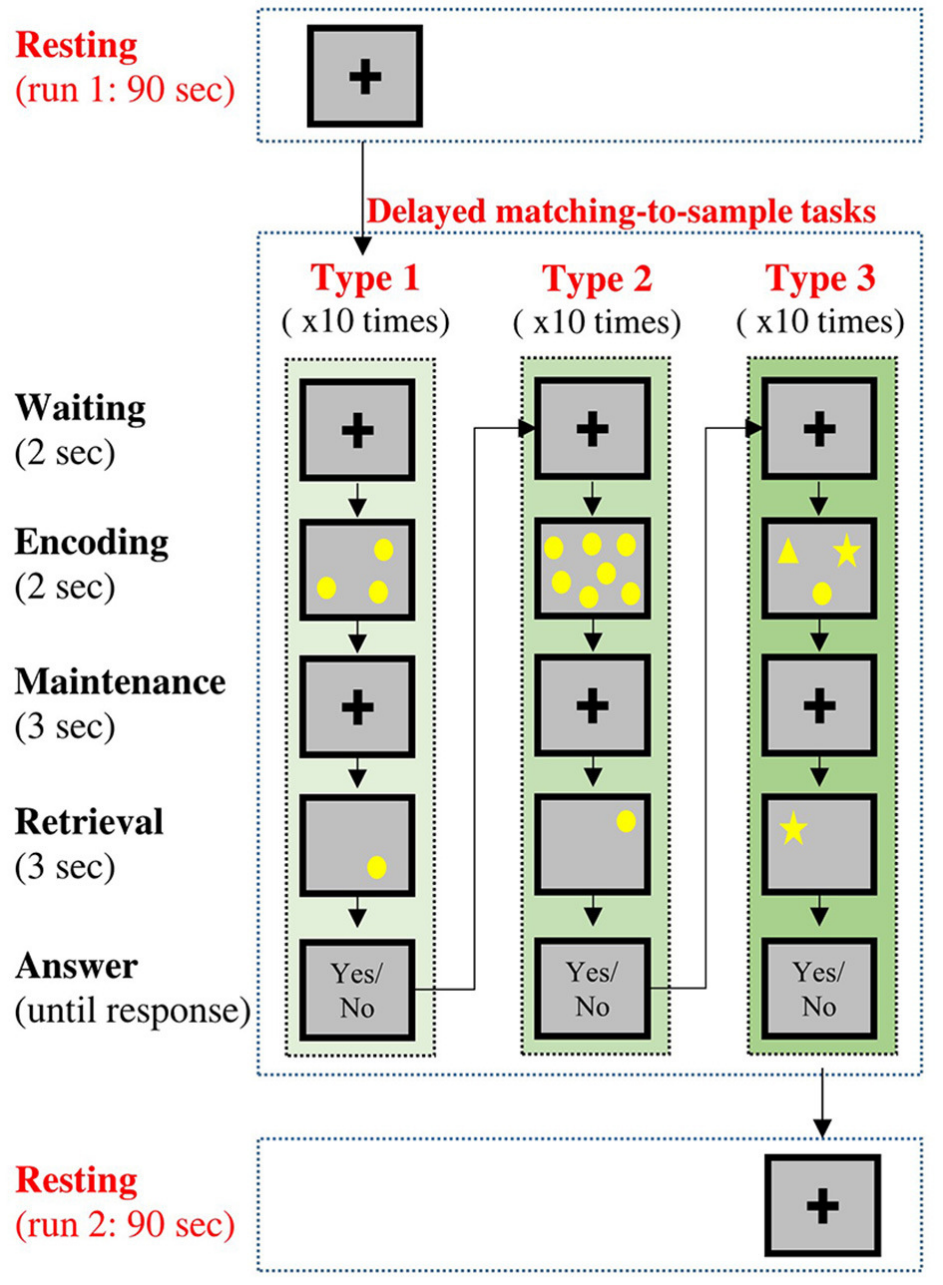
Temporal sequence of the experimental procedures. Each participant would undergo two resting-state conditions and three delayed matching-to-sample (DMTS) tasks. Type 1 and Type 2 tasks requires the participants to remember the locations of stimuli, and Type 3 task requires the participants to remember both the contents and locations of stimuli.

In this experiment, all the participants underwent two sessions of resting-state condition (named resting run 1 and resting run 2) along with a working memory condition between two resting-state conditions (Figure 1). During each resting-state condition (90 s), the participants were instructed to gently keep their eyes fixated on a central fixation cross without doing/thinking anything purposefully. During the memory condition, the participants performed three types of delayed DMTS tasks (Sahakian et al., 1988; Fowler et al., 1995), with 10 trials for each type.

A DMTS trial included three phases. In the encoding phase (2 s), a set of sample stimuli was presented on the screen for the participants to remember. In the maintenance phase (3 s), the corresponding visual display was removed from the screen, and the participants were required to keep the information in their working memory. In the retrieval phase (3 s), a question display was presented on the screen, and the participants were required to judge if the contents of the question display match (both in terms of shape and position) those in the sample display. The participants were instructed to answer the question with a button press after the retrieval phase without a time limit. The three types of DMTS tasks varied in terms of contents to be remembered: 1. Type 1: the participants were required to remember the locations of three circles randomly placed on the screen, 2. Type 2: the participants were required to remember the locations of seven circles randomly placed on the screen. 3. Type 3: the participants were required to remember the locations of three different shapes (a circle, a square, and a star) randomly placed on the screen.

### EEG Acquisition and Preprocessing

**Figure 2:**
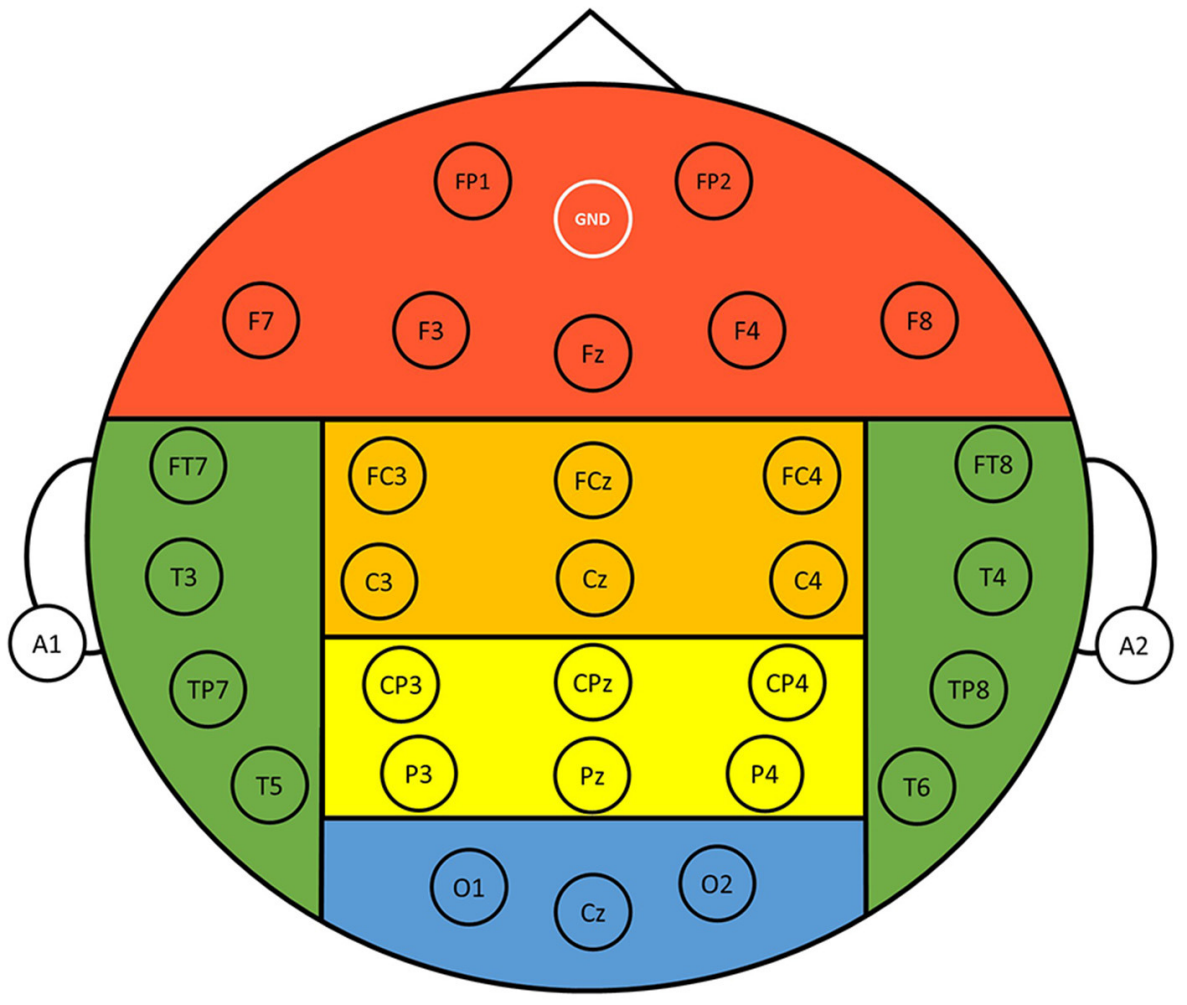
Layout of the 30 recording electrodes. The positions of the electrodes follow the International 10-20 system. References were at A1 and A2 positions, and the ground electrode was at the forehead (GND). The entire scalp region was divided into six different regions for analysis, namely, frontal (FP1, FP2, F3, F4, F7, F8, Fz), central (FC3, FC4, FCz, C3, C4, Cz), parietal (CP3, CP4, CPz, P3, P4, Pz), occipital (O1, O2, Cz), left temporal (FT7, T3, TP7, T5), and right temporal (FT8, T4, TP8, T6) regions.

EEG signals were recorded with a 33-channel Quick-Cap connected to a 40-channel NuAmps (NeuroScan Amplifier, Compumedics Inc., Charlotte, NC, USA). The layout of the electrodes followed the International 10–20 system (Figure 2), where A1 and A2 were reference electrodes, the ground channel was at the forehead, and the remaining 30 electrodes were used for recording EEGs. Impedance was kept below 10 kOhm by applying Electro-Gel (Compumedics Inc., Charlotte, NC, USA) to the electrodes. Ocular activity (i.e., electrooculography, EOG) was monitored with two electrodes placed above the left eye and the right side of the right eye, respectively. The recorded EEG and EOG signals were amplified and filtered (0.5–100 Hz), and then digitized with a sampling rate of 500 Hz using the NuAmp amplifier from NeuroScan Inc. Ocular artifacts coming from blinking or eye movements were removed from the EEG signals using the artifact removal software from NeuroScan (Scan4.5). Afterward, the EEG signals were further filtered using a Finite Impulse Response (FIR) filter (0.5–50 Hz). Finally, other possible artifacts caused from generic discontinuities and electromyography were removed using the independent component analysis (ICA) and ADJUST algorithm (Mognon et al., 2011) provided in the EEGLAB (Infomax ICA).

### Feature Extraction: Between-Run Similarity Based on Spectral Powers

The purpose here was to quantify the intra-subject variability of the EEG signals between the two resting runs using a between-run similarity (BRS) of spectral powers. The calculation of the BRS consists of five steps.

Step 1: segmentation of the resting-state EEG signal into epochs

For each participant and for each run of resting state, the 90-s EEG signal was segmented into 36 epochs of 6-second length, and there is overlap of 60% between two consecutive epochs. Then, we visually inspected all the segmented EEG epochs to make sure that the data used for later analysis were noise- and artifact-free. Among the 74 participants, nine (three with AD, four with MCI, two HCs) had only 35 clean epochs for analyses in both runs or in one of the two runs. The rejected epochs had large-amplitude peaks in voltage, which could be due to some technical issue during recordings. Data of the remaining 65 participants were all clean (i.e., all 36 epochs per run were used for later analyses).

Step 2: calculation of band power for each epoch

For each participant, spectral band powers of delta (1–4 Hz), theta (4–8 Hz), low alpha (8–10 Hz), high alpha (10–13 Hz), low beta (13–20 Hz), high beta (20–30 Hz), and gamma (30–45 Hz) were extracted from each epoch using fast Fourier Transform (FFT). Considering a specific band, let $BP_{ij}^{r}$ be the band power of the *i*th epoch recorded from the *j*th electrode of a specific scalp region, where *r* ∈ {1, 2} denotes the *r*th run of the resting state, and, for example, *j* = 1, 2, …, 7 for the frontal region. Note that *n* = 35 for some of the 74 participants (the nine aforementioned), and *n* = 36 for the remaining 65 participants.

Step 3: computation of the average power vector for each scalp region

Then, for each band and for each run, we extracted the averaged powers of each 6-s epoch across the electrodes in a given scalp region,

(1) $$BP_{i}^{r}=\frac{1}{n_{e}}\sum_{j=1}^{n_{e}}BP_{ij}^{r}$$

where *n*_*e*_ is the number of electrodes in the scalp region of interest, and *i* denotes the *i*th epoch. Thus, for a given scalp region, the average scalp powers of the seven different bands corresponding to the *i*th epoch were then concatenated to form a power vector ${\text{p}}_{i}^{r}$ of dimension 7, where *r* denotes the run number of resting-state EEG recordings (1 or 2 in this case).

Step 4: calculation of between-run similarity for each participant and each scalp region

The aim here is to calculate the similarity between the EEG power vectors of the two runs for each scalp region and for each participant. Supposing that *s*_*ij*_ denotes the similarity between the average scalp powers of the *i*th (*i* = 1, …, *n*) and *j*th (*i* = 1, …, *m*) epochs in the 1^st^ and the 2^nd^ run of the resting-state EEGs, the similarity can be measured by the Euclidean distance as

(2) $$s_{ij}=\frac{1}{\left\|p_{j}^{2}-p_{i}^{1}\right\|}$$

A higher value of *s*_*ij*_ corresponds to a higher between-run similarity between the vectors ${\text{p}}_{i}^{1\;}$and ${\text{p}}_{j}^{2}$. Then, calculating the similarities between all possible pairs of ${\text{p}}_{i}^{1}$ and ${\text{p}}_{j}^{2}$ and then averaging all the similarities will yield the averaged between-run similarity for a specific scalp region,

(3) $$S=\frac{1}{n\times m}\sum_{i=1}^{n}\sum_{j=1}^{m}s_{ij}$$

Step 5: standardization

The value of *s*_*ij*_ could be very small, because the distance between vectors ($\left||{\text{p}}_{j}^{2}-{\text{p}}_{i}^{1}|\right|$) is considerably large in most cases. As a result, the value of the between-run similarity could approach to zero. Therefore, for the *i*th participant (*i* = 1, 2, …, 74), we further standardize his/her BRS *S*(*i*) using the HC group as the benchmark,

(4) $$S\left(i\right)=\frac{S\left(i\right)-mean\;\left(HC\right)}{std\;\left(HC\right)}$$

where *mean* (*HC*) and *std* (*HC*)stand for the mean and standard deviation of the between-run similarity values calculated from the HCs, respectively. There are two primary reasons behind performing standardization in this study. First, since the raw BRS values are generally small, standardization will help zoom in on the potential differences, if any, between groups. Second, in the field of clinical science, a common approach to quantitatively evaluate the level of dysfunction or impairments of individuals with clinical diagnosis is to perform standardization based on data from the healthy population (e.g., IQ, depression levels, cognitive declination, etc.). Accordingly, we apply the same concept to perform the study-driven standardization of the task-induced BRS based on data from HCs in the same study. In other words, data from healthy controls is treated as a distribution reference for estimating how far the task-induced BRS of individuals with MCI or AD deviates from the healthy population.

After performing the above five steps, six BRS values (i.e., six scalp regions) for each of the 74 participants were obtained. Each BRS represents the task-induced intra-subject variation of resting-state EEG power in a specific scalp region. A high between-run similarity corresponds to a low intra-subject variation between the two separate runs of resting states.

### Classification

Two commonly used classifiers were adopted for classification, a linear discriminant analysis (LDA) and a support vector machine (SVM) classifier. LDA finds a linear decision boundary in the original space of patterns to separate classes. Its decision function is given by

(5) $$\begin{array}{l} D_{LDA}\left(x\right)={\left(\mu_{P}-\mu_{N}\right)}^{t}\Sigma^{-1}x-\\ \;\frac{1}{2}{\left(\mu_{P}-\mu_{N}\right)}^{t}\Sigma^{-1}\left(\mu_{P}+\mu_{N}\right)-\ln\left(\frac{C_{P}\pi_{N}}{C_{N}\pi_{P}}\right),\; \end{array}$$

where **x** ∈*R*^*d*^ is test data, *t* denotes the transpose of a matrix, μ_*P*_, and μ_*N*_ are is the mean vectors of the training data of the positive and negative classes, respectively, Σ is the covariance matrix of the training data, *C*_*P*_ and *C*_*N*_ are the penalty weight for the positive and negative classes, respectively, and π_*P*_ and π_*N*_ are the *a priori* probabilities of the positive and negative classes, respectively. Here, the penalty weights for both classes were set the same, i.e., *C*_*P*_ = *C*_*N*_. Note that the feature dimension of the data *d* (*d* ∈ [1, 6]) represents how many between-run similarity features are used. For example, *d* = 6 if the between-run similarities of all the six scalp regions are used as the features, and *d* = 1 if only a between-run similarity of the scalp regions is adopted as the feature for classification.

SVM maps the training data ${\left\{\left({\text{x}}_{i},y_{i}\right)\right\}}_{i=1}^{L}$, *y*_*i*_ ∈ {−1, +1} are is class labels, into a higher-dimensional feature space from the original space *R*^*d*^ *via* a non-linear mapping φ, and then finds a hyperplane **w**^*t*^φ(**x**) + *b* = 0, which maximizes the margin of separation and minimizes the training errors, formulated as

(6) $$\begin{array}{l} \text{ Minimize }\frac{1}{2}{\left\|w\right\|}^{2}+C\sum_{i=1}^{N}\xi_{i}\\ subject\;to\;y_{i}\left(w^{t}\varphi \left(x_{i}\right)+b\right)-1+\xi_{i}\geq 0\;\forall i\\ \;\xi_{i}\geq 0\;\forall i\; \end{array}$$

where **w** and *b* are the weight vector and the bias of the SVM hyperplane, respectively, ξ_*i*_ is slack variables representing the error measures of training data points, and *C*is a penalty weight. For an unseen data **x**, its class label is predicted by the decision function

(7) $$D_{SVM}\left(x\right)=\sum_{x_{i}\in SV}\alpha_{i}y_{i}K\left(x_{i},x\right)+b,$$

where α_*i*_ are is Lagrange multipliers [obtained by solving the dual problem of (6)], SV denotes the set of support vectors (the training data points whose Lagrange multipliers satisfying 0 < α_*i*_ ≤ *C*), and *K* is the kernel function. In this study, the radial basis function (RBF) function $K\left(x_{i},x\right)=\exp(-\gamma {\left\|x_{i}-x\right\|}^{2})$ was chosen as the kernel, where γ is the kernel parameter. The optimal value of the bias *b* can be determined by the Kuhn–Tucker condition. The test data **x** is classified as positive if *D*_*SVM*_(**x**) > 0; negative otherwise.

### Performance Evaluation and Parameter Optimization

After performing feature extraction, we obtained 74 data (74 vectors) from the 74 participants, and each data consists of *d* between-run similarity values from *d* different scalp regions. Although the main goal of this study was to examine the feasibility of using between-run similarity features to achieve promising MCI-HC classification performance, we still performed three different binary classification tasks (AD vs. MCI, MCI vs. HC, and AD vs. HC) to see if such intra-subject variability could contribute to the classification between AD and MCI or between AD and HC.

Similar to previous EEG studies, the number of available EEG data in this study is limited, mainly because the time for recruiting participants was rather long. Performing the usual 10- or 5-fold cross validation is not appropriate, because the number of test data used for testing in each fold is considerably small: one misclassified set of data will result in a large error rate in each fold. Therefore, following the previous studies (Liao et al., 2017; Wu et al., 2018), LOPO-CV was adopted as the performance evaluation method to test the participant-independent accuracy, which predicts how well the results of the proposed method will generalize to unseen data. Take the classification of MCI (24 participants) vs. HC (27 participants) as an example. In each fold of LOPO-CV, data (*d*-dimensional vectors) from 50 participants were used to train the classifier, and then the *d*-dimensional data from the remaining participant served as the test data. This step was repeated until the data of every participant had been used as test data once. We then recorded the classification accuracy, computed as the number of correctly classified participants divided by the total number of participants from two groups. Hereafter, the classification accuracy or accuracy would be used to refer to those obtained by the LOPO-CV procedure.

Both the proposed between-run similarity feature and the LDA classifier involve no free parameter. SVM involves two parameters (*C* and γ). We optimized the parameters of SVM using the LOPO-CV and grid search methods. The values of *C* and γ were searched in the same set {2^−29^, 2^−27^, …, 2^27^, 2^29^}, leading to 961 parameter grids. The best parameter grid results in the highest classification accuracy.

### Feature Selection

The question now is how to determine the best feature subset to gain the highest classification accuracy. In other words, the goal is to determine the optimal value of the feature dimension *d*: which combination of the between-run similarities of the scalp regions is the best for classification. To this end, we adopted a commonly used wrapper-based feature selection method—the sequential forward selection (SFS) algorithm (Guyon and Elisseeff, 2003).

Let *N*_*s*_ be the number of scalp regions (*N*_*s*_ = 6). The optimal feature selection procedure based on the SFS algorithm initially finds the best single between-run similarity feature of a scalp region, which gives the highest LOPO-CV classification accuracy. It is noted that here the LOPO-CV was performed on the data of the participants from two groups, as mentioned in section Performance Evaluation and Parameter Optimization (e.g., 51 data in MCI vs. HC). Subsequently, *N*_*s*_ − 1 pairs of features of the scalp regions are formed by combining each of the remaining features of the scalp regions with the best single feature, and the best pair (i.e., the pair that gives the highest LOPO-CV classification accuracy) is selected. Following the same logic, *N*_*s*_ − 2 triples of features are formed using each of the remaining features of the scalp regions and the best feature pair, and the best triple is selected (i.e., the triple that gives the highest LOPO-CV classification accuracy). This procedure is repeated until all the *N*_*s*_ features are tested. Finally, the best feature set is the one resulting in the highest LOPO-CV classification accuracy. In other words, we can rank these six features from the best to the worst after this SFS-based procedure. The top-*n*-ranked features giving the highest LOPO-CV classification accuracy form the optimal feature subset, where 1 ≤ *n* ≤ *N*_*s*_.

**Figure 3:**
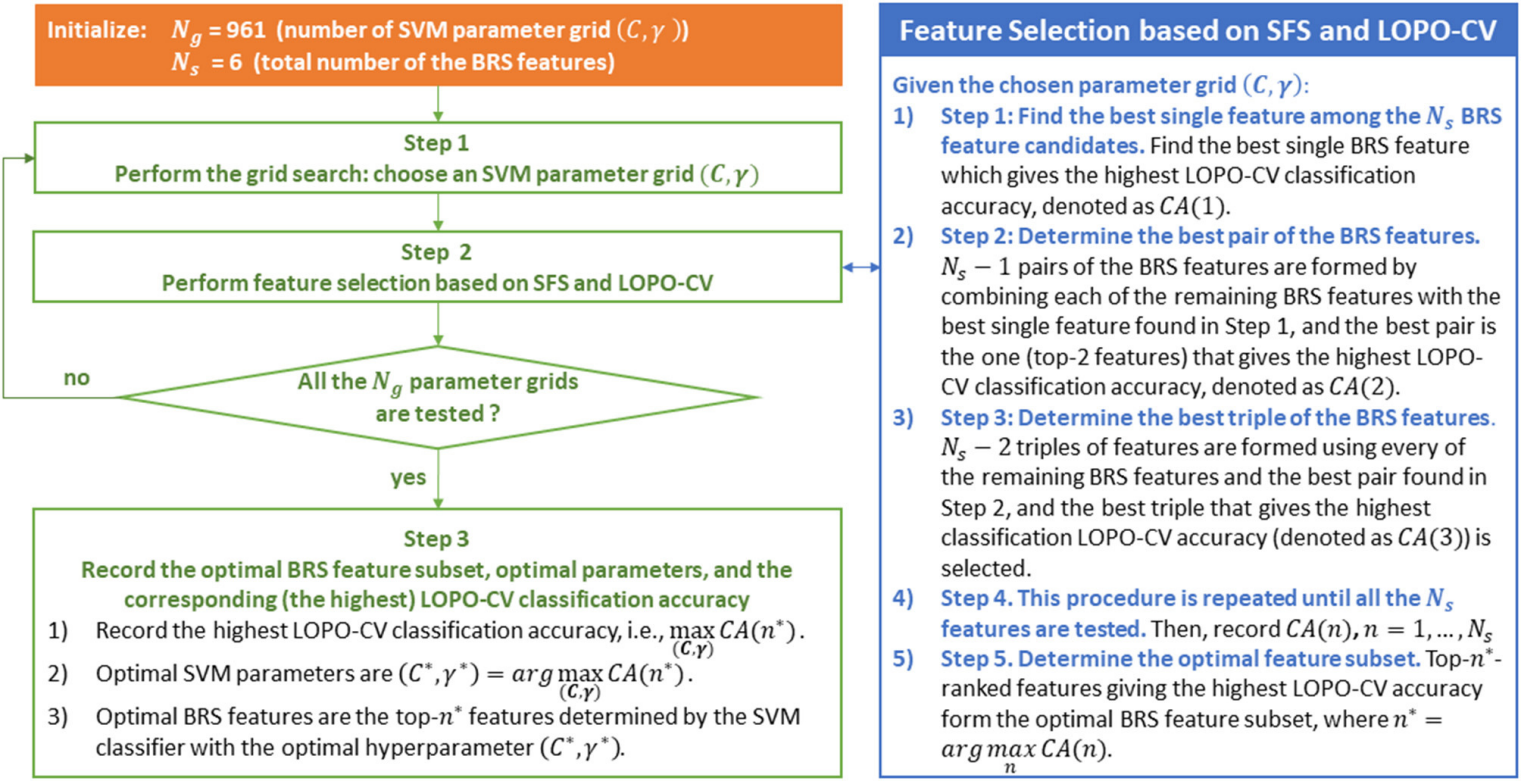
SFS-based between-run similarity (BRS) feature selection and the grid-search-based parameter determination procedure based on the use of an SVM classifier.

SFS is a wrapper-based greedy approach for feature selection, which has the advantage of achieving better accuracy than filter-based feature selection approach, but with the disadvantage of being more time-consuming (Guyon and Elisseeff, 2003). Fortunately, the number of the BRS feature candidates is only six, and thus the SFS algorithm used in this study is not computationally expensive. Wrapper approaches include the interaction between feature subset and classification model (Saeys et al., 2007). In other words, wrapper-based methods are classifier-specific in which the methods search for the best subset of features that optimizes the generalization classification accuracy of a chosen classifier (Kudo and Sklansky, 2000), and the generalization performance used for evaluating the features is often estimated by *k*-fold cross validation or LOPO-CV (Wu et al., 2018). Therefore, for the classifiers LDA and SVM, the optimal BRS feature subset selected by the SFS method could be different, as presented in the results (**Figure 6**). Even using the same SVM classifier, the optimal feature subset selected by the SFS algorithm could also be different for different SVM parameters (different values of *C* and γ), because the generalization performance of SVM varies with the parameter grid (*C*, γ). In summary, for the SVM classifier, the SFS-based feature selection and the grid-search-based parameter determination must be carried out together. The combination of the optimal BRS feature subset and the SVM classifier with the optimal hyperparameter gives the highest LOPO-CV classification accuracy. The SFS-based feature selection and the grid-search-based parameter determination procedure are summarized in Figure 3.

As illustrated in this figure, LOPO-CV is performed to estimate the generalization performance under the condition that a parameter grid and a feature subset have been given in advance. The calculated classification accuracy based on LOPO-CV is subsequently used for evaluating the chosen parameter grid and the feature subset. In other words, in each fold of the LOPO-CV process in this study, the test data (a BRS feature vector) from one participant is involved in the feature selection and the parameter optimization procedure. Accordingly, the test data in this LOPO-CV process are, in fact, validation data, not independent test data.

### Statistical Analysis

Since the data of between-run similarities did not pass the Kolmogorov–Smirnov test, we performed the Wilcoxon rank sum test to statistically test three pre-planned between-group comparisons: AD vs. HC, MCI vs. HC, and AD vs. MCI. Since each between-group comparison included six tests (data of 6 scalp regions), we used a Bonferroni corrected α level of 0.0083 (0.05/6) to correct for multiple comparison.

## Results

### Behavior Performance Among Groups in Different DMTS Tasks

**Figure 4:**
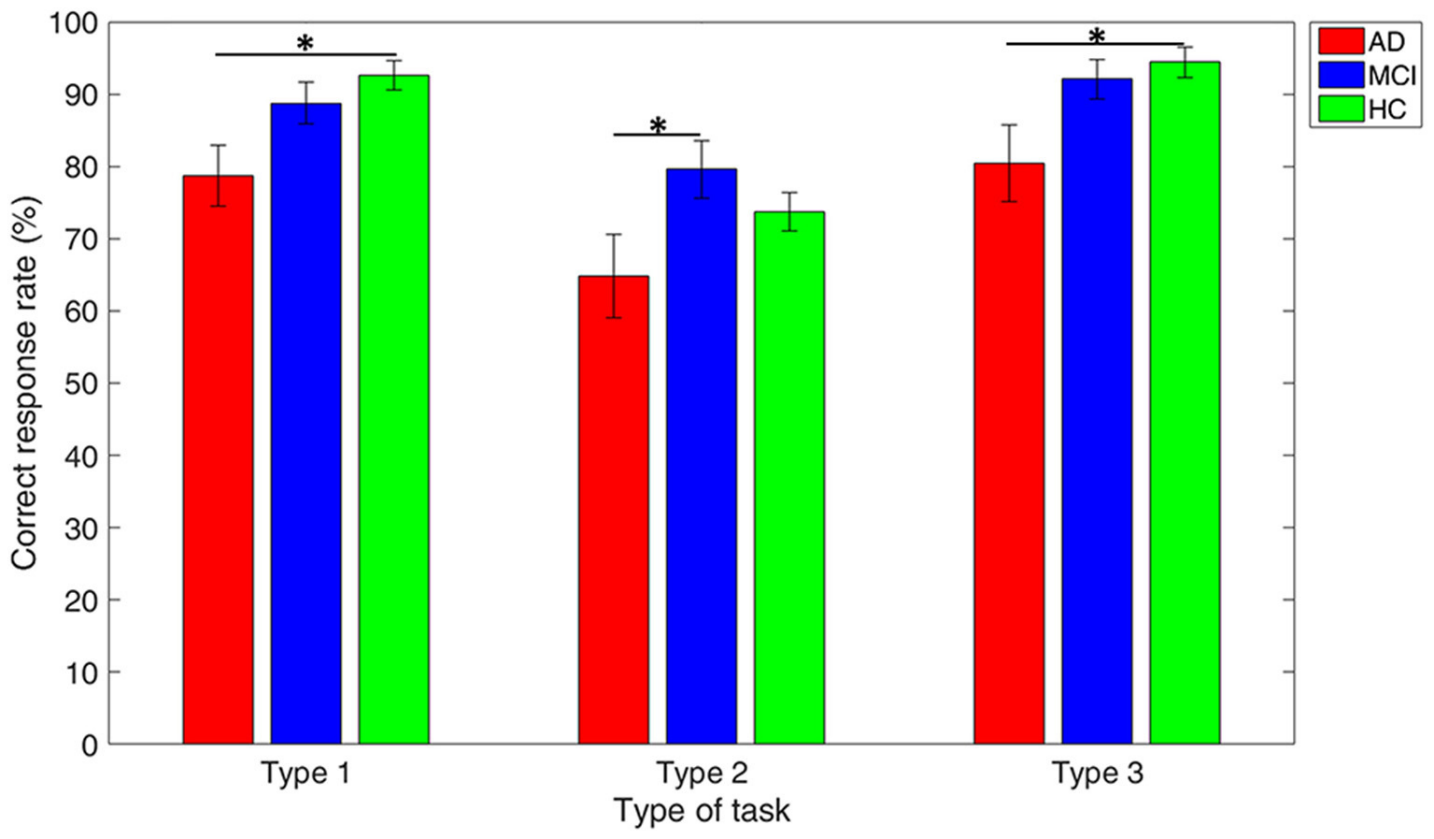
Performance comparison among groups in each type of working memory tasks. Note: error bars indicate standard errors of the means of the correct response rate and * refers to *p* < 0.05.

Figure 4 reveals that the mean accuracy of DTMS tasks gradually increases from AD, MCI, to HC, except in Type 2 of the DMTS task where the MCI group showed higher mean accuracy than the HC group. All the three groups showed worst performance in Type 2 working memory task, as compared with the other two types. The between-group comparison in each type of working memory tasks revealed a significant difference in accuracy between the AD vs. HC group in Type 1 (*p* = 0.003) and Type 3 (*p* = 0.012), and AD vs. MCI group in Type 2 (*p* = 0.038).

### Comparing the Between-Run Similarities Among the HC, MCI, and AD Groups

**Figure 5:**
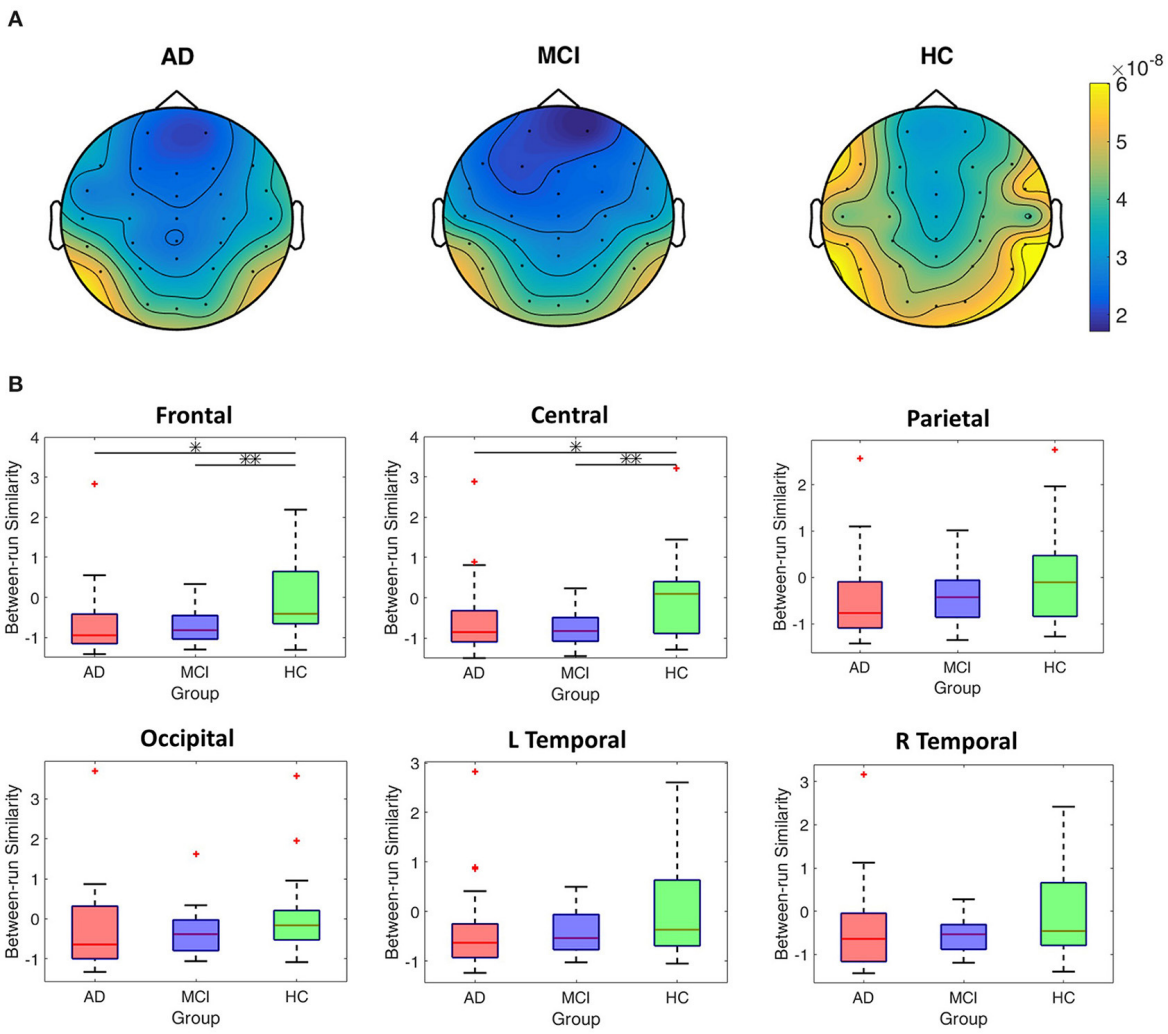
Comparisons of between-run similarities across the AD, MCI, and HC groups. **(A)** The topographic distribution of the non-standardized between-run similarities of resting-state EEGs for each group. **(B)** Boxplots of the standardized between-run similarities for each group in different scalp regions. *p < 0.05; **p < 0.05/6.

Figure 5A shows the EEG scalp topography of between-run similarities (non-standardized) of resting-state EEGs in each group. The HC group showed the highest between-run similarity over all the scalp regions. To further compare the group difference in between-run similarities, we perform statistical tests on the standardized between-run similarity (see Method for detailed calculation) on each scalp region across all groups. As shown in Figure 5B, the HC group shows the highest median values of between-run similarities (standardized) among the three groups at all the scalp regions (Figure 5B). However, there was significant group difference in the between-run similarity only in the frontal (*p* = 0.006, Bonferroni corrected alpha = 0.0083) and central (*p* = 0.004, Bonferroni corrected alpha = 0.0083) scalp regions in the MCI vs. HC comparison.

**Figure 6:**
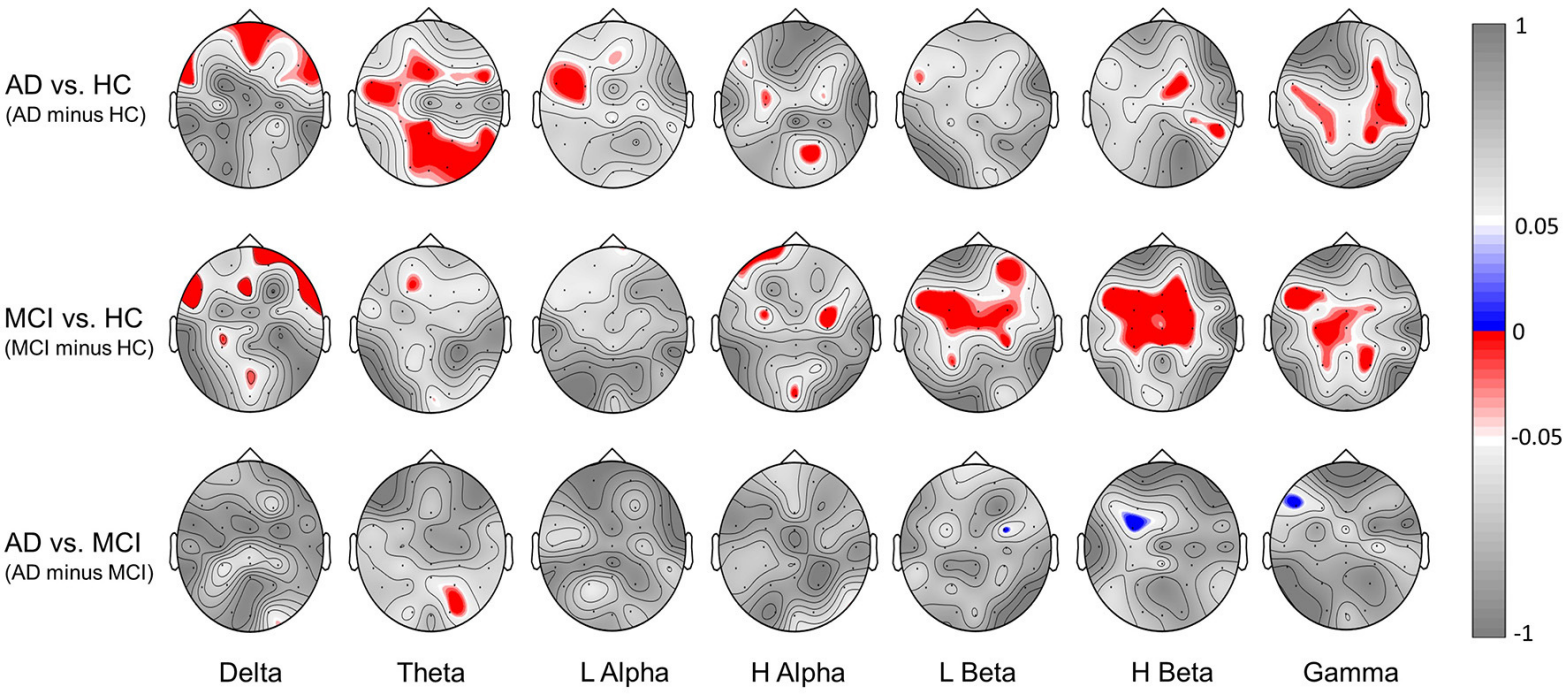
Significant group difference in spectral power-based BRS values within individual bands (delta, theta, low alpha, high alpha, low beta, high beta, gamma) for the comparison of [AD minus HC], [MCI minus HC] and [AD minus MCI]. Here we showed the topography of *p*-values; red indicates a negative difference, and blue indicates a positive difference.

Figure 6 further illustrates the significant group difference in spectral power based BRS within each individual band (delta, theta, low alpha, high alpha, low beta, high beta, gamma) across the whole scalp region for the comparison of AD vs. HC, MCI vs. HC, and AD vs. MCI. The results show that the task-induced intra-subject variability of resting-state EEG is larger (lower BRS values) in the MCI group than the HC group over the frontal, central, and parietal scalp regions in low-beta, high-beta, and gamma bands; larger in the AD group than the HC group over the frontal, parietal, and occipital scalp regions in the delta and theta bands. Furthermore, in line with the results shown in Figure 5A, almost no statistically significant difference is found between the AD and MCI groups. These findings indicated that both low and high frequencies contribute to spectral power-based BRS difference between AD vs. HC or between MCI vs. HC but with different topographic distributions.

### Comparing the Classification Accuracies Between the Three Binary Classifications

Classification results of the three binary classification tasks are shown in Figure 6, where for each classification task the SFS method was used to determine the best between-run similarity feature subset. The highest classification accuracy for the three classifications of AD vs. HC, MCI vs. HC, and AD vs. MCI, was 74.47, 80.39, and 78%, respectively, and these results were all achieved by SVM. The results indicate that the between-run similarity can be a good candidate to classify between different groups, especially between MCI vs. HC. Moreover, when the SVM classifier was used, the highest accuracy 80.39% of the MCI-HC classification was achieved by only one feature (the between-run similarity of the parietal scalp region that includes six electrodes). Similarly, the highest accuracy of 74.47% of the AD-MCI classification was achieved when only one between-run similarity feature extracted from the four electrodes of the left temporal region was used. Considering the feasibility in the context of community healthcare, both classification accuracy and usability are critical. A small number of electrodes can largely shorten the time needed for preparation. Therefore, the results demonstrate the high usability of the proposed between-run similarity feature in both MCI-HC and AD-MCI classifications.

### Comparing Classification Performances Between Other Features Extracted From Single-Run Resting-State EEGs and Between-Run Similarity Features

We further compared the classification performance of the proposed between-run similarity feature with other widely used features extracted from single-run (1^st^ run) resting-state EEGs in different scalp regions in the three binary classification problems, namely, spectral power (SP), complexity, and connectivity features. Here, a simple LDA classifier was employed.

Fractal dimension (FD) has been widely used for measuring the complexity of an EEG signal. The FD of a signal can be estimated by different methods, such as those of Katz's and Higuchi's methods and the correlation dimension. Katz's FD (KFD) (Katz, 1988) has been a widely accepted approach, because it involves no free parameter and is, therefore, computationally cheap. Also, it is less sensitive to noise in comparison with the Higuchi's FD (Esteller et al., 2001), and has recently shown its high sensitivity to the change of in a mental state in various BCI applications, for example, the EEG-based detection of concentration level (Yeh et al., 2018). Coherence, a measure for the synchrony between two electrodes' EEG signals of two electrodes at a specific frequency band or point, is frequently- applied as an EEG connectivity feature (Liao et al., 2017). The feature extraction procedures for the three types of features to be compared are as follows.

- Spectral power (SP). Take theta SP of the frontal region as an example. For each electrode, we first calculated the theta band power values from the *n* EEG epochs of the 1^st^ run resting-state EEGs separately, and then averaged the *n* values. The seven average theta power features from the seven frontal electrodes were sent to the classifier for classification (i.e., *d* = 7).
- Katz's FD (KFD). As an example, the frontal-region KFD was calculated with the following steps: 1) for each electrode, we calculated the KFD values from the *n* EEG epochs of the 1^st^ run resting-state EEGs separately; 2) then we averaged the *n*KFD values across epochs; 3) finally the seven averaged KFD features from the seven frontal electrodes were sent to the LDA classifier.
- Coherence (Coh). Take delta-band coherence in the frontal region as an example. For each pair of electrodes, we calculated the delta-band coherence values from the *n* EEG epochs of the 1^st^ run resting-state EEGs separately, and then averaged the *n* values to obtain an average coherence feature. Since there were seven electrodes in the frontal region, we obtained totally $\frac{7\times \left(7-1\right)}{2}=21\;$ coherence features of delta band, which form a 21-dimensional feature vector fed into the LDA classifier.

The results based on spectral power, KFD, and coherence features are listed in Table 2.

**Table 2:** Comparison of classification accuracies between different features and scalp regions using LDA classifier (in %).

**Features**		**Frontal**	**Central**	**Parietal**	**Occipital**	**Left temporal**	**Right temporal**
AD vs. HC	δ (SP/Coh)	58/58	52/**64**	46/54	38/36	**60**/**60**	44/58
	θ (SP/Coh)	**64**/**62**	56/54	56/38	**60**/**62**	54/**62**	50/**62**
	Lα(SP/Coh)	54/54	56/**64**	52/**62**	54/50	**60**/**64**	48/46
	Hα(SP/Coh)	58/**60**	54/**60**	54/54	48/48	58/58	50/**64**
	Lβ(SP/Coh)	58/**60**	54/52	44/52	54/40	58/48	50/48
	Hβ(SP/Coh)	**62**/58	**60**/52	50/52	48/42	**60**/52	44/56
	γ (SP/Coh)	**64**/52	44/34	42/42	42/42	**62**/38	46/54
	KFD	56	46	50	44	56	54
MCI vs. HC	δ (SP/Coh)	50.98/43.14	54.90/52.94	54.90/45.10	35.29/35.29	45.10/45.10	47.06/58.82
	θ (SP/Coh)	49.02/58.82	45.10/52.94	**62.75**/56.86	37.25/31.37	54.90/**60.78**	47.06/29.41
	Lα(SP/Coh)	47.06/41.18	43.14/45.10	54.90/43.14	45.10/43.14	52.94/50.98	54.90/37.25
	Hα(SP/Coh)	56.86/47.06	50.98/43.14	47.06/56.86	39.22/49.02	43.14/50.98	50.98/47.06
	Lβ(SP/Coh)	56.86/49.02	58.82/**60.78**	47.06/**62.75**	47.06/**62.75**	58.82/43.14	43.14/54.90
	Hβ(SP/Coh)	**64.71**/47.06	**60.78**/54.90	52.94/50.98	39.22/39.22	52.94/**64.71**	49.02/39.22
	γ (SP/Coh)	**64.71**/49.02	52.94/31.37	47.06/45.10	45.10/52.94	**62.75**/52.94	52.94/50.98
	KFD	58.82	56.86	54.90	50.98	56.86	43.14
AD vs. MCI	δ (SP/Coh)	36.17/46.81	44.68/55.32	40.43/46.81	36.17/31.91	40.43/57.45	57.45/36.17
	θ (SP/Coh)	55.32/46.81	59.57/59.57	**61.70**/53.19	44.68/51.06	57.45/53.19	42.55/53.19
	Lα(SP/Coh)	**61.70**/**62.34**	53.19/44.68	48.94/44.68	36.17/**62.34**	34.04/**61.70**	29.79/46.81
	Hα(SP/Coh)	**61.70**/46.81	**61.70**/34.04	53.19/31.91	31.91/61.70	38.30/42.55	34.04/59.57
	Lβ(SP/Coh)	46.81/51.06	57.45/42.55	48.94/38.30	38.30/**62.34**	34.04/48.94	29.79/59.57
	Hβ(SP/Coh)	40.43/55.32	55.32/48.94	48.94/38.30	40.43/51.06	31.91/59.57	36.17/**61.70**
	γ (SP/Coh)	38.30/40.43	40.43/31.91	44.68/34.04	42.55/59.57	42.55/48.94	44.68/**62.34**
	KFD	44.68	46.81	57.45	44.68	48.94	40.43

*Spectral power and coherence features are based on different frequency bands, namely, delta (δ), theta (θ), low alpha (Lα), high alpha (Hα), low beta (Lβ), high beta (Hβ), and gamma. LOPO-CV classification accuracies higher than or equal to 60% were in boldface*.

The spectral powers did not show satisfactory performance for any of the three classification problems. The highest accuracies for the AD-HC, MCI-HC, and AD-MCI classifications were 64, 64.71, and 61.7%, respectively, which were all slightly higher than the chance level (50%). Similar accuracies were also found for coherence. The best accuracies for the three binary classifications (AD-HC, MCI-HC, and AD-MCI), for example, were 64 (central), 64.71 (left temporal), and 62.34% (frontal). Compared with spectral power and coherence features, KFD performs relatively worse. All the accuracies from KFD were close to the chance level (< 60%).

**Figure 7:**
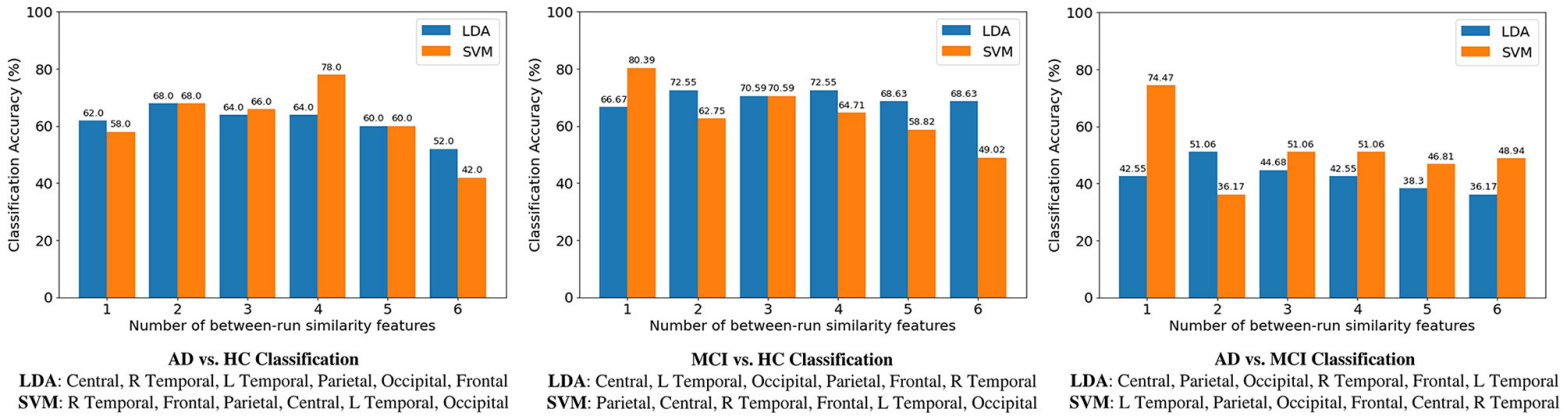
Comparison of accuracies among three binary classifications. For each classification, the sequential forward selection (SFS) algorithm was used the find the optimal feature subset. Take AD-HC classification as an example. The best feature subset contains four features (the between-run similarities of right temporal, frontal, parietal, and central scalp regions) when a support vector machine (SVM) was used as the classifier. Accuracy decreases to 42% if all the six between-run similarity features were used, i.e., without feature selection.

By further comparing the LDA-based results of the between-run similarity features shown in Figure 7, where the best accuracies are 68 (AD vs. HC: two features), 72.55 (MCI vs. HC: two features), and 51.06% (AD vs. MCI), we can see that the between-run similarity feature outperforms the three types of widely used features extracted from single-run resting-state EEGs in both the AD-HC and MCI-HC classifications.

## Discussion

In the brain-computer interface (BCI) community, intra-subject variability has been a challenge to be overcome. However, in this study, we have shown that in terms of classifying between individuals with MCI and healthy one, the intra-subject variability could be an advantage instead. We therefore proposed the between-run similarity feature to represent the task-induced intra-subject variability of the EEGs recorded in two separate runs of resting-states, before and after a challenging working memory task (i.e., the DMTS). The primary goal of this study was not to propose a novel EEG feature that can perform better than any other existing features but to propose a novel feature extraction framework by which the extracted feature (i.e., the tasked-induced intra-subject variability) can provide more discriminative information for identifying individuals with MCI, and perform better than the usual architecture of feature extraction from single-run resting-state EEGs. The results have demonstrated that the between-run similarity feature is indeed able to achieve high classification performance, especially in the MCI vs. HC classification (80.39%). It is believed that the accuracy can be further improved by combing the proposed between-run similarity feature and other features that showed encouraging performance [e.g., multiscale entropy (MSE) Maturana-Candelas et al., 2019; Sun et al., 2020].

Previously, variants of the DMTS tasks have been tested in few recent EEG-based MCI studies. These few studies focused statistical analysis on the task-related EEG (EEGs recorded during performing the task) event-related potentials (e.g., Li et al., 2017). Task-related EEGs may contain discriminative information for classification. However, task-related EEGs sometimes suffer from signal contamination issue from great EOG/EMG artifacts because of excessive eye and body (especially the neck and facial actions) movements, which are not easy to remove completely. In contrast, resting-state EEGs are less likely to be contaminated by artifacts, and thus relatively easy to implement in clinical practice. The proposed feature extraction framework records resting-state EEGs before and after the DMTS-based working memory task and extract task-induced intra-subject variability features. This “hybrid” approach is unique, because it not only preserves the advantage of the resting-state EEGs (i.e., cleaner signals), it also capitalizes on the clinical traits of working memory dysfunction of the MCI group, which is presumably more informative.

Few recent studies have also used spectral features to characterize MCI, such as band power (absolute power) (Rabbani et al., 2016; Ruiz-Gómez et al., 2018a; Kashefpoor et al., 2019) and relative power (Musaeus et al., 2018; Farina et al., 2020). The reported accuracies in these studies ranged between 60 and 80%. It is a bit unfair to compare the accuracy of the between-run similarity feature with the accuracies reported in those bodies of literature, because there were differences in terms of experimental settings as well as the inclusion and exclusion criteria of participants. Nevertheless, based on the same participants and settings, the results have indicated that, for both the MCI-HC and AD-HC classifications, the spectral power-based between-run similarity is superior to the spectral power feature extracted from a single run resting-state EEGs.

Working memory performance is an important indicator for evaluating memory ability. DMTS task has been largely used to evaluate working memory ability in animals, such as pigeons (Case et al., 2015; Zentall and Smith, 2016), monkey (Pontecorvo and Evans, 1985), and hens (Foster et al., 1995). In human participants, DMTS was also used to study working memory performance in alcohol-dependent (Bowden et al., 1992) and nicotine-dependent individuals (Janes et al., 2013). In addition, the voltage peak-based qEEG ratio of posterior parietal to the dorsolateral prefrontal cortex (DLPFC) extracted from task-induced EEG signals based on DMTS showed high performance for classification between normal aging individuals and patients with mild AD (94% specificity and 88% sensitivity) (Sneddon et al., 2005). As expected, this study demonstrated that working memory performance gradually decreases from the HC to MCI to AD group, except in the Type 2 task. All the three groups performed poorly in the Type 2 task, likely because of the larger memory loads of the Type 2 task (participants need to remember the locations of seven circles). A DMTS task with heavy memory loads can lead to decreases in accuracy (Adamson et al., 2000).

As Figure 5 reveals, the lower between-run similarities in the AD and MCI groups than the HC group at the frontal and central scalp regions suggest a more noticeable difference between resting-state EEGs recorded before and after a challenging working memory task in the AD and MCI groups. The higher task-induced intra-subject variability suggests that performing cognitively exhausting working memory tasks causes greater disturbance to the degenerated brains in individuals with MCI or AD (Kirova et al., 2015), which then leads to greater difficulties to restore the same resting state as measured before performing the task. A useful analogy is comparing the difference of heart rate variability (HRV) or rhythm of breath during resting state before and after a 5K jogging between individuals with cardiovascular dysfunction and normal people. Although the spatial resolution of EEG makes it difficult to measure the exact signal source, the current frontally oriented results may very likely reflect the common findings on the critical role of prefrontal cortices in working memory and executive function (Guntekin et al., 2008; Papadaniil et al., 2016; Jiang et al., 2021).

The general patterns of lower between-run similarities in the AD and MCI groups than the HC group (Figures 5A, 6) seems to also echo the observation of lower connectivity across electrodes in the AD and MCI groups than the HC group based on single-run resting-state EEGs (Michels et al., 2017; Ruiz-Gómez et al., 2018a,b). For example, results from cross-sample entropy-based connectivity (Ruiz-Gómez et al., 2018b) revealed that both the AD and MCI groups showed an overall lower electrode-to-electrode connectivity than the HC group in the frequency band of 14–19 Hz, which is very close to the low beta band in this study. Both the loss of electrode-to-electrode connectivity in previous studies and the decrease in task-induced intra-subject variation (i.e., a decreased capability to maintain stable resting-state EEG patterns after cognitively exhausting tasks) in this study could be associated with the reduction in cortical-cortical connections or gray matters observed in the brains of individuals with AD or MCI (Jeong, 2004; Maestu et al., 2021) compared to the HC group. Future studies combining structural and functional brain scans (e.g., voxel-based morphometry, white matter fiber tracking, or functional connectivity) and EEG recordings will be required to verify this link.

Somewhat counter-intuitively, the binary classification accuracy is higher for the MCI vs. HC classification than for the AD vs. HC classification. This result actually echoes the statistical results showing that the between-run similarity values over the frontal and central scalp regions were significant between MCI and HC but not between AD vs. HC. A possible explanation may attribute to the observation that some patients with AD were not able to keep up with the DMTS tasks because of high difficulties. Thus, the after-task resting EEGs could be quite similar to the before-task resting EEGs in these patients with AD, because they simply did not spend too much effort on the tasks, as compared with the MCI and HC group. This speculation could be partially supported by the significantly lower DMTS performance for the AD group as compares with the HC (Types 1 and 3) and MCI (Type 2) groups.

If we assumed that MCI was simply a mild AD, then the difference between them could follow a somewhat linear gradient from mild to moderate to severe in terms of the severity degrees of dementia. If this was true, it would be reasonable to predict that the classification problem of AD vs. MCI vs. HC may be more easily solved: there could exist a single EEG feature that covaries with severity degree, which can then be used for assessing cognitive dysfunction degree, with a higher degree corresponding to a higher probability/risk to be AD. Another possibility would be that there exists a feature set such that in the feature space, the class separability between AD and HC will be larger than that between MCI and HC. However, the results from previous studies were controversial and do not support the assumption above. Although some previous results have shown that AD-MCI classification accuracy is higher than the MCI-HC classification accuracy (Ieracitano et al., 2019; Meghdadi et al., 2021), some other studies have reported the opposite (Sharma et al., 2019) or similar (Fiscon et al., 2018; Ieracitano et al., 2020) result patterns. The reported result in this study shows that MCI-HC (80.39%) is slightly higher than AD-HC (78%) in classification. However, such an accuracy drop of 2.3% reflects only one more misclassified participant in the LOPO-CV process, because in the AD-HC classification, misclassified data in a testing fold of the LOPO-CV resulted in an increase in error rate (1/50 = 2%). Accordingly, in this study, the classification results on AD vs. HC and MCI vs. HC may be also viewed as similar.

From the view of psychopathology, there is no clear evidence either to assume that MCI and AD can be viewed as a gradient change or severity along the same clinical trait. First, according to the diagnostic criteria based on NINCDS-ADRDA and DSM-5, MCI is defined as a distinct syndrome of abnormal cognitive change deviating from the normal aging process, but is not grounded to dementia. In other words, MCI is not considered as early dementia (Bruscoli and Lovestone, 2004). Second, MCI in 32% of individuals developed into AD at the 5th-year follow-up, as aforementioned. In other words, MCI in about 70% of individuals would not process to become AD in 5 years. In contrast, many healthy elderly people develop AD directly without going through the MCI stage in clinical practice, meaning that the MCI stage is not necessarily the only transient state between healthy conditions and AD. Although MCI has a relative high risk of developing into AD, it is not the only risk factor. Other risk factors for AD include hypertension, type 2 diabetes mellitus, dyslipidemia, cardiovascular defects, and alteration of the apolipoprotein E4 (Livingston et al., 2020). All this evidence may help explain why AD-HC classification accuracy is not necessarily higher than the MCI-HC classification.

Being the first one to apply intra-subject variability in EEGs as features for the classification between AD/MCI vs, HC, this study suffers several limitations that can be further addressed in the near future. First, there are many different similarity-based measures. Although the Euclidean distance is a straight forward approach for measuring signal similarity, its application in high-dimensional data is more limited (Grootendorst, 2021). It may be possible to improve classification performance further using other types of similarity measures. For example, in a recent study, Hellinger distance and Bhattacharyya distance showed their effectiveness with highly noisy EEG signals (Chen G. et al., 2020). Although comparisons between different approaches of similarity measures are beyond the scope of this study, they certainly merit attention in evaluating the effectiveness of using different between-run similarity measures as neurophysiological features for classifying neurodegenerative diseases. Second, to be able to build a reliable classification model, we would certainly need a much larger sample size, especially in the context of clinical practices. Therefore, future studies with a much larger sample size would be necessary to further test the validity and reliability of task-induced intra-subject variability for the classification between AD, MCI, and HC groups.

Last but not least, the core concept behind this novel feature extraction framework is highly flexible to be integrated with other types of EEG features, complexity feature for example. In this study, since we hoped to focus the investigation on whether the new framework may lead to an improvement in the MCI-HC classification, we, therefore, decided to implement it with the more typical spectral power features and test the effectiveness of the spectral power-based BRS. On another note, other types of EEG feature can also be applied to quantify the signal similarity between the two separate runs of resting-state EEGs, as long as we replace the spectral powers with other EEG features in steps 2–4 in the calculation of the BRS. However, such a comparison is beyond the scope of this study. Nevertheless, based on the results of this study, it is expected that the task-induced intra-subject variability based on other types of features could also perform better than single-run resting-state EEGs and achieve even higher classification performance. In the future, further investigation on intra-subject variability based on other features may provide additional insights into how the EEG dynamics of individuals with MCI would change before and after performing working memory tasks (e.g., loss of complexity or irregularity in EEGs).

## Conclusion

This study investigated the value of using intra-subject EEG variability between two separate runs of resting states, before and after a sequence of challenging working memory tasks, as a feature for the classification between individuals with MCI vs. healthy controls. We derived a between-run EEG power similarity as a measure of the intra-subject variability, and applied the machine learning methods (SFS-based feature selection and SVM classification) to determine the most sensitive scalp regions for classification. The main findings are 2-fold. First, the between-run similarity provided encouragingly high LOPO-CV classification accuracy (~80%) for the MCI-HC and AD-HC classifications, and such performance was superior to the spectral power features extracted from single-run resting-state EEGs. Second, the feature selection results suggested that the 80% MCI-HC classification accuracy could be achieved using an SVM classifier and the six electrodes over the parietal scalp region. Moreover, the results were obtained by LOPO-CV. Because of the small EEG dataset, the LOPO-CV process was performed together with the feature selection and the parameter determination procedure. Follow-up studies will be needed to test the proposed methods on an independent dataset to further examine its generalization performance. Indeed, the intra-subject variability has been a challenging issue in terms of stability of BCI application. In contrast and counter-intuitively, the results reveal that the intra-subject variability between two resting-state EEG data collected before and after a challenging memory task can actually be a promising approach for MCI-HC classification. This study, therefore, shed new light on how we may transform the disadvantage of intra-subject variability into an advantage in the field of BCI application.

## Data Availability Statement

The datasets presented in this article are not readily available because the current patient dataset cannot be shared in any form due to regulation of IRB and Personal Information protection Act. Requests to access the datasets should be directed to Yi-Hung Liu, lyh@mail.ntust.edu.tw.

## Ethics Statement

The studies involving human participants were reviewed and approved by Institutional review board of Taipei Veterans General Hospital (IRB No: 2017-06-009A). The patients/participants provided their written informed consent to participate in this study.

## Author Contributions

T-TT participated in the algorithm design, EEG data recording, analysis, and drafting. C-FT participated in the study conception, participant recruitment, EEG data collection, and discussion. Y-TH helped process the EEG data and participated in the result analysis. C-YL participated in the analysis and discussion in critical points. C-TW conceptualized the study design, designed the memory task experiment, coordinated data collection, involved in data analyses, and participated in manuscript writing and revision. Y-HL was responsible for project coordination, conceptualized the study design, secured the researching funding, designed analyses protocols, and participated in paper writing and revision. All authors contributed to the article and approved the submitted version.

## Conflict of Interest

The authors declare that the research was conducted in the absence of any commercial or financial relationships that could be construed as a potential conflict of interest.

## Publisher's Note

All claims expressed in this article are solely those of the authors and do not necessarily represent those of their affiliated organizations, or those of the publisher, the editors and the reviewers. Any product that may be evaluated in this article, or claim that may be made by its manufacturer, is not guaranteed or endorsed by the publisher.
